# First-Principles Prediction of High and Low Resistance States in Ta/h-BN/Ta Atomristor

**DOI:** 10.3390/nano14070612

**Published:** 2024-03-30

**Authors:** Lan He, Shuai Lang, Wei Zhang, Shun Song, Juan Lyu, Jian Gong

**Affiliations:** 1 School of Physical Science and Technology, Inner Mongolia University, Hohhot 010021, China; 2 State Key Laboratory of Superlattices and Microstructures, Institute of Semiconductors, Chinese Academy of Sciences, Beijing 100083, China

**Keywords:** interface barrier, band gap states, atomristors

## Abstract

Two-dimensional (2D) materials have received significant attention for their potential use in next-generation electronics, particularly in nonvolatile memory and neuromorphic computing. This is due to their simple metal–insulator–metal (MIM) sandwiched structure, excellent switching performance, high-density capability, and low power consumption. In this work, using comprehensive material simulations and device modeling, the thinnest monolayer hexagonal boron nitride (h-BN) atomristor is studied by using a MIM configuration with Ta electrodes. Our first-principles calculations predicted both a high resistance state (HRS) and a low resistance state (LRS) in this device. We observed that the presence of van der Waals (vdW) gaps between the Ta electrodes and monolayer h-BN with a boron vacancy (V_B_) contributes to the HRS. The combination of metal electrode contact and the adsorption of Ta atoms onto a single V_B_ defect (Ta_B_) can alter the interface barrier between the electrode and dielectric layer, as well as create band gap states within the band gap of monolayer h-BN. These band gap states can shorten the effective tunneling path for electron transport from the left electrode to the right electrode, resulting in an increase in the current transmission coefficient of the LRS. This resistive switching mechanism in monolayer h-BN atomristors can serve as a theoretical reference for device design and optimization, making them promising for the development of atomristor technology with ultra-high integration density and ultra-low power consumption.

## 1. Introduction

Nonvolatile resistive switching, also known as memristor, has emerged as an important concept in the development of high-density information storage, computing, and neuromorphic systems [1,2,3,4,5,6]. Memristors typically have a vertical metal–insulator–metal (MIM) sandwich structure, with the insulator serving as the active layer. Traditional active layer materials include bulk metal oxide films, such as HfO_2_ [7,8], Al_2_O_3_ [9], and TiO_2_ [10,11]. However, in recent years, with the rapid development of integrated circuits, significant efforts have been made to decrease the insulator thickness in order to improve storage density and reduce power consumption [12,13,14,15,16]. Studies have shown that atomically thin two-dimensional (2D) materials, such as transition metal dichalcogenides (TMDs) [17,18,19,20,21,22], hexagonal boron nitride (h-BN) [23,24,25,26], and other recently developed 2D materials [26,27,28], exhibit the resistance switching phenomenon, rendering them a bright application prospect in high-performance memristor devices. In particular, atomristors, which feature nonvolatile resistive switching in atomically thin 2D materials, have drawn much attention due to their nanometer-thin insulating layers, forming-free characteristics, high on/off resistance ratio, and low set/reset voltages [21,23,27]. The flat surface of 2D materials is beneficial for regulating defect engineering and facilitating the formation of conductive filaments, thereby reducing the randomness and variability in device performance. The discovery of nonvolatile resistance switching in 2D monolayer materials has added a new class of materials and dimensions to consider for memory storage, offering the potential for size scaling. 

However, the physical mechanism of the conductive channel in a monolayer 2D material atomristor is still under strong debate. Significant research is needed to understand the detailed switching process and improve reliability [29]. Among all 2D materials, h-BN is considered an ideal resistance switching material due to its wide band gap, excellent thermal and chemical stability [30,31,32]. It has been reported that the nonvolatile resistance switching mechanism in monolayer h-BN atomristors is dominated by the adsorption and desorption process of metal atoms on atomic sheets [23]. However, the density of states suggests that a very high defect concentration (up to 25%) is needed to achieve a large enough on/off ratio, making it difficult to fully explain the resistance switching phenomenon in these devices. Additionally, the weak van der Waals (vdW) interaction can provide more degrees of freedom for lattice matching between electrode metals and monolayer h-BN. Previous studies have demonstrated that the resistance characteristics of atomristors vary significantly when using different electrodes on the monolayer h-BN [23,24,33]. Therefore, a more comprehensive understanding of the different resistance switching behaviors in monolayer h-BN with various electrodes is required. 

In this work, we utilized density functional theory (DFT) simulations and the non-equilibrium Green’s function (NEGF) method [34] to investigate the conductive mechanism of the Ta/h-BN/Ta atomristor. Our findings suggest that the switching process in this device is primarily influenced by the presence of boron vacancy (V_B_) defects. We propose that under the influence of an electric field, the Ta atom can dissociate from the metallic surface and adsorb above the V_B_ defect (Ta_B_). Due to the ultra-thin thickness of the h-BN monolayer, Ta atoms can migrate and penetrate through the h-BN, forming a full-chain conductive filament through the V_B_ defects. Our results reveal that the combined effect of interface contact and atomic defects plays a crucial role in the resistance switching mechanism of Ta/h-BN/Ta atomristors, providing new opportunities for boosting the performance of 2D atomristors.

## 2. Computational Methods

The simulations in this study were performed using the first-principles software package QuantumATK, which is based on DFT theory in combination with NEGF methods [34]. The exchange–correlation potential was described by the generalized gradient approximation (GGA) of the Perdew–Burke–Ernzerhof (PBE) functional, and the wave function was expanded using the PseudoDojo basis for all atoms. A cut-off energy of 75 Hartree was applied for the plane wave expansion of valence electron wave functions. The Monkhorst–Pack k-point samplings for the calculations of electronic structures and quantum transport properties were 12 × 12 × 1 and 4 × 4 × 133, respectively. All structures were optimized after adding a 20 Å vacuum thickness to avoid periodic image interactions. The nudged elastic band (NEB) method was utilized to determine the migration barrier for metal atoms [35]. The convergence criteria for energy and force in structure optimization and barrier calculation were set to 1 × 10^−4^ eV and 0.05 eV/Å, respectively. The current can be calculated using the Landauer–Bűttiker formula [36]:

(1) $$I=\frac{2e}{\hbar }\int_{-\infty }^{+\infty }T\left(E,V_{\mathrm{bias}}\right)\;\left[f(E-E_{\mathrm{FL}})-f(E-E_{\mathrm{FR}}))\right]\;dE$$

where *T*(*E*,*V*_bias_) is the transmission coefficient at a given bias voltage (*V*_bias_), *f*(E) is the Fermi–Dirac distribution function, and *E*_FL/FR_ are the Fermi energies of the left and right electrodes.

## 3. Results and Discussion

**Figure 1:**
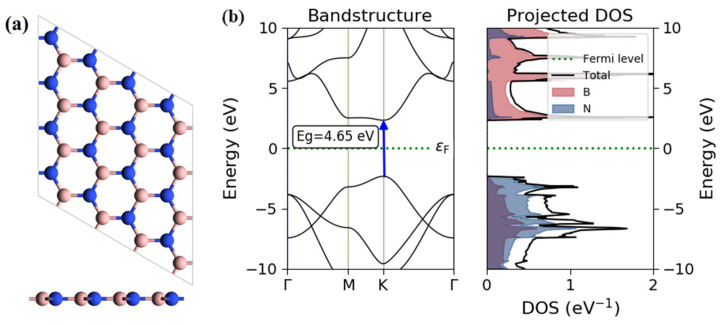
(**a**) Top and side views of monolayer h-BN; the pink and blue spheres represent B and N atoms, respectively. (**b**) Electronic band structure and density of state of monolayer h-BN; the Fermi level is set to 0 eV.

Monolayer h-BN has a hexagonal honeycomb structure, as shown in Figure 1a, with an optimized lattice parameter of 2.52 Å. The calculated band structure and density of state (DOS) in Figure 1b indicate that monolayer h-BN is an insulating material with a direct band gap of 4.65 eV.

**Figure 2:**
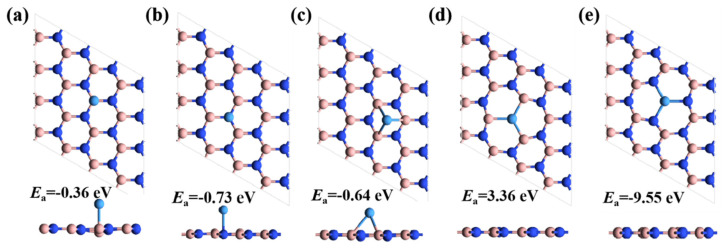
Adsorption of Ta atoms on various sites of the intrinsic monolayer h-BN, including on the top of (**a**) boron atoms, (**b**) nitrogen atoms, and (**c**) honeycomb. Adsorbed states of Ta atoms on defective h-BN with (**d**) V_N_ and (**e**) V_B_. Both side and top views are shown for each case.

To systematically estimate the influence of adsorption, we investigated the structures and adsorption energies of Ta atoms in different sites/defects of monolayer h-BN. The adsorption energy (*E*_a_) was calculated using the following formula:

(2) $$E_{a}=E_{\mathrm{total}}-E_{\mathrm{Ta}}-E_{\mathrm{BN}}$$

where $E_{\mathrm{total}}$ is the total energy of a Ta atom adsorbed on intrinsic or defective h-BN, $E_{\mathrm{BN}}$ is the energy of intrinsic or defective h-BN, and $E_{\mathrm{Ta}}$ is the energy of an isolated Ta atom. Figure 2a–c show different adsorption positions on intrinsic monolayer h-BN. It is clear that Ta atoms can only be weakly adsorbed on monolayer h-BN, with *E*_a_ values ranging from −0.36 to −0.73 eV. In addition, in Figure 2d, we can observe that the Ta atom has a large positive adsorption energy in the nitrogen vacancies (V_N_) with an *E*_a_ of 3.36 eV, indicating that it cannot provide an active site to form a stable conductive channel. On the contrary, the adsorption of Ta atoms on boron vacancies (V_B_) is much more stable, with an energy of −9.55 eV, as shown in Figure 2e. These adsorption energies demonstrate that Ta atoms tend to bind with the V_B_ defect, providing the possibility of forming conductive channels for resistance switches.

**Figure 3:**
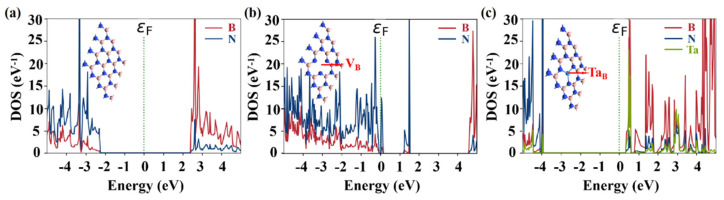
Partial density of states (PDOS) of monolayer h-BN under different conditions. (**a**) Intrinsic, (**b**) V_B_, and (**c**) Ta_B_, which refers to a Ta atom adsorbed on V_B_.

Figure 3a–c show the partial density of states (PDOS) for both defect-free and defective h-BN. A comparison with intrinsic h-BN reveals that the introduction of V_B_ results in p-type doping of monolayer h-BN, as evidenced by the valence band maximum (VBM) approaching the Fermi level (ε_F_) and a few band gap states appearing in the forbidden band. Conversely, the presence of the Ta_B_ defect in h-BN leads to n-type doping, with the conduction band minimum (CBM) approaching the Fermi level and a few band gap states appearing at the edge of the conduction band.

**Figure 4:**
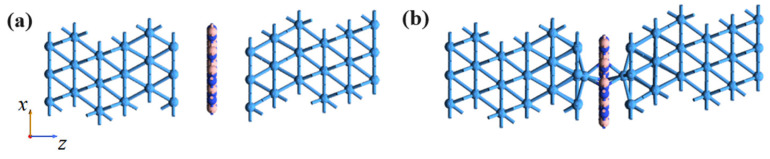
Two distinct interface structures between Ta and a monolayer h-BN. (**a**) Ta/V_B_/Ta and (**b**) Ta/Ta_B_/Ta, which refers to Ta atoms attached to the V_B_ defect.

On the other hand, the interface contact properties between metal Ta and monolayer h-BN play an important role in 2D atomristor devices. Firstly, we created two heterojunction interfaces consisting of Ta metal and monolayer h-BN. As shown in Figure 4a,b, the metal Ta is in contact with h-BN on its (111) surface. In a real situation, the contact metal consists of multiple layers. To accurately model this, the bulk metals extend to the sixth layer, and the third to sixth layers from the interface are set as rigid body constraints. This allows for the relaxation of the h-BN monolayer and the first to second metal layers. In this heterojunction, the lattice constant of Ta is adjusted to match that of h-BN, resulting in a mean strain of 0.13%. We assume that V_B_ defects are naturally present in h-BN materials, which has been supported by experimental reports [37,38] and the observed forming-free characteristics in 2D atomristors [18,23]. In our study, we considered the V_B_ defect to be inherent, as shown in Figure 4a, where the metal Ta/monolayer h-BN with a V_B_ defect forms a weak vdW contact, with an interfacial distance of 3.82 Å. However, in Figure 4b, the metal Ta atoms tend to interact with nearby V_B_ defects and become adsorbed on them, forming chemical bonds with the three surrounding N atoms. Since the LRS of an atomristor relies on the formation of conductive atomic filaments, we predicted that the adsorption of Ta atoms above V_B_ (Ta_B_) can be utilized as the possible conductive points, while the vdW gaps between Ta and h-BN can be utilized as the initial HRS.

**Figure 5:**
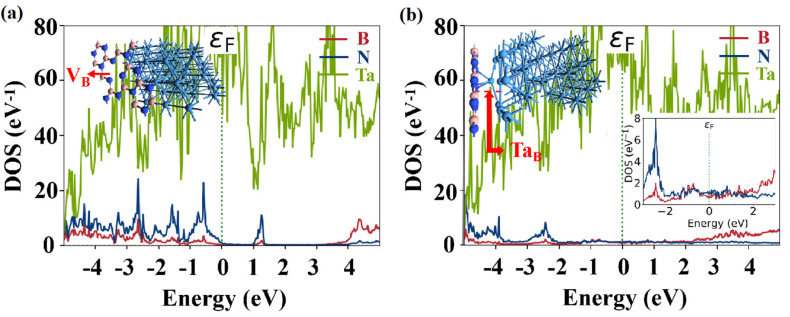
PDOS of Ta/h-BN heterostructures: (**a**) Ta/V_B_ and (**b**) Ta/Ta_B_ interfaces. The *ε*_F_ is located at zero energy.

Furthermore, a Schottky barrier (SB) generally exists in the metal/semiconductor interface, which is closely related to the electron injection efficiency [39,40,41]. The *SB* height at contact interface is defined as

(3) $$\Phi_{SB,n}=E_{CBM}-\varepsilon_{F}$$

(4) $$\Phi_{SB,p}=\varepsilon_{F}-E_{CBM}$$

where $\varepsilon_{F}$ is the Fermi level, and *E_CBM_* and *E_VBM_* are the *CBM* and *VBM* of the semiconductor, respectively. A commonly used method for evaluating the interface *SB* heights is through PDOS calculations, which involve determining the energy difference between the $\varepsilon_{F}$ and the *CBM* or *VBM* of the contacted semiconductor. To better understand the interface contact properties before and after the switching event, we compared the PDOS of Ta/h-BN heterostructures. In Figure 5a, which shows a vdW-contacted model, it is evident that the interface between metal Ta and h-BN with V_B_ defect forms a p-type Ohmic contact. Additionally, a few band gap states are formed within the band gap of h-BN. However, in Figure 5b, it can be observed that the band gap of monolayer h-BN was significantly altered and many band gap states are formed by the Ta_B_ contact, indicating a metallic-like behavior near the Ta absorption sites. This suggests that carrier injection at the Ta_B_ interface is barrier-free in the vertical direction. Therefore, these band gap states may play a crucial role as occupied states during the electron tunneling process and should not be disregarded in the modeling of Ta/h-BN/Ta atomristors.

**Figure 6:**
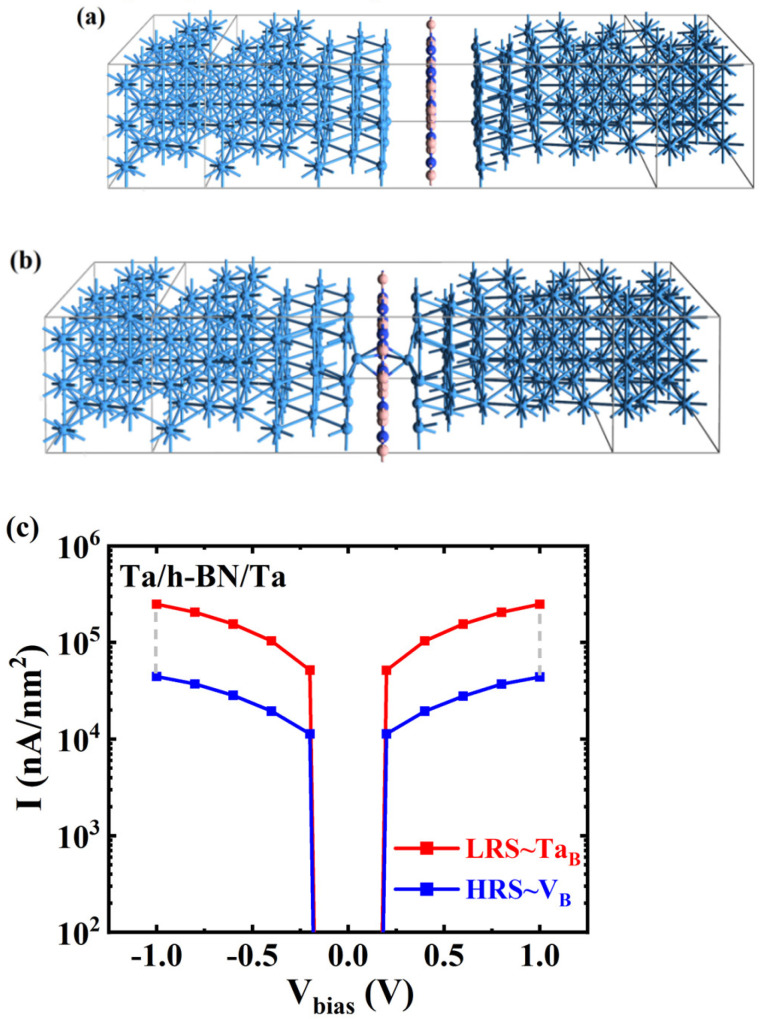
Device structures of the Ta/h-BN/Ta atomristor (**a**) without a conductive channel and (**b**) with a full-chain conductive filament, where Ta atoms are adsorbed on the V_B_ defect. (**c**) The calculated *I*-*V* curves for these two device structures.

To further study the resistance mechanism of monolayer h-BN atomristor, the transport characteristics of h-BN atomristor were calculated. The Ta/h-BN/Ta atomristors with Ta electrodes were simulated as a whole by using a two-probe model and the DFT theory coupled with NEGF. In our study, we considered the V_B_ defect to be inherent, as shown in Figure 6a. Under the influence of an electric field, the Ta atom can dissociate from the metal electrode surface and adsorb above the V_B_ defect. Furthermore, due to the ultra-thin thickness of h-BN monolayer (~0.33 nm), Ta atoms can migrate under the electric field and penetrate through the h-BN atomic layer, forming a full-chain conductive filament through the V_B_ defects, as depicted in Figure 6b. We refer to this process as the dissociation–adsorption–permeation process. To verify the interface contact and atomic defect related conductive mechanism, the current–voltage (*I*-*V*) output characteristic curve of the device was simulated using the NEGF-DFT method. The results in Figure 6c demonstrate that the Ta/V_B_/Ta configuration maintains insulating properties with low currents. This is due to the vdW gaps between the Ta electrodes and h-BN monolayer, which create an energy barrier for vertical electronic emission, resulting in the device being initially in the HRS. On the other hand, the defective configuration with Ta atoms adsorbed on V_B_ shows a higher current, with the current signal reaching up to one orders of magnitude higher than that of the HRS (e.g., 3.37 × 10^3^ vs. 2.05 × 10^5^ nA/nm^2^ at 1.0 V). These results demonstrate that the atomic filaments formed by Ta_B_ defects serve as ideal conductive channels in the Ta/h-BN/Ta atomristor. Moreover, the current switching ratio calculated here is comparable to the experimental measurement in Ni/h-BN/Ni devices [24], but lower than that of Au/h-BN/Au and Ag/h-BN/Ag devices [23,33]. Therefore, further clarification of the conductive mechanism in the h-BN atomristor is necessary, in order to effectively regulate device performance.

**Figure 7:**
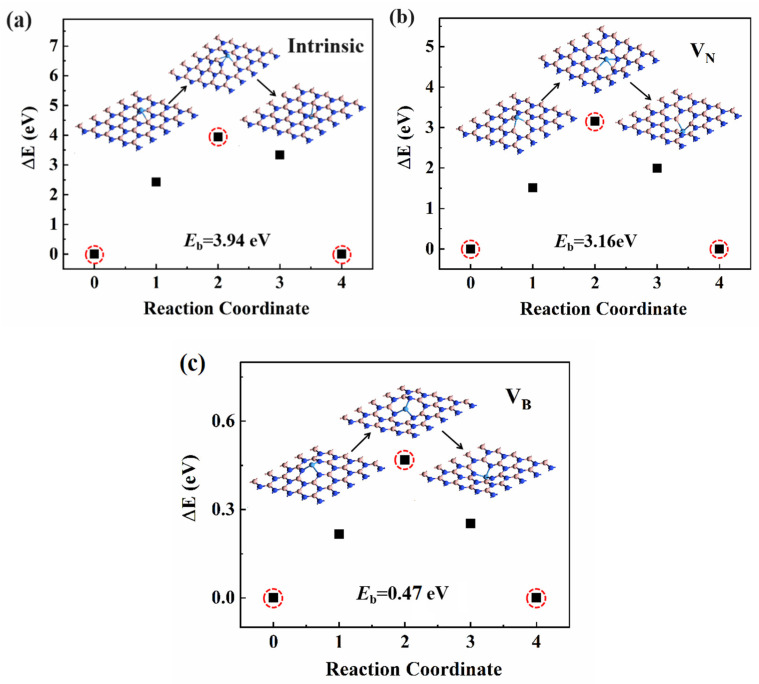
(**a**) Energy barrier for Ta atoms passing through (**a**) intrinsic h-BN (**b**) via the V_N_ defect and (**c**) V_B_ defect. The red circles indicate the initial, barrier, and final states; the corresponding structures are shown in the insets.

In the actual switching process, Ta atoms tend to migrate from one electrode to the opposite electrode in the direction of the applied electric field. However, this process may not be easily observed through experimental methods. Therefore, to determine the feasibility of this migration process, the energy barrier (*E*_b_) was calculated using the nudged elastic band method. As shown in Figure 7, we calculated the potential barrier that Ta needs to overcome to pass through the h-BN monolayer under different conditions. In Figure 7a, it can be seen that in the absence of defects, the *E*_b_ required for Ta atoms to pass through h-BN is as high as 3.94 eV, indicating that a full-chain conductive channel cannot be formed through a defect-free h-BN layer. However, in the presence of defects, as shown in Figure 7b,c, the *E*_b_ for Ta atoms penetrating h-BN through V_N_ is 3.16 eV, while in the case of V_B_, the *E*_b_ is only 0.47 eV. This suggests that the Ta atoms can penetrate through the monolayer h-BN via V_B_ and form a full-chain conductive filament in the Ta/h-BN/Ta atomristor. Therefore, our current calculation of the LRS adopts the structure of two Ta atoms adsorbed on both sides of V_B_ in h-BN, as shown in Figure 6b. This phenomenon has also been observed in previous research, supporting our prediction [21,23,24,25,33].

**Figure 8:**
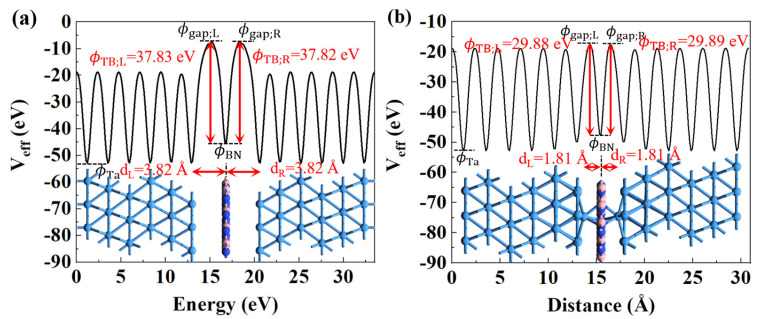
Plots of the effective potential (*V*_eff_) for the Ta/h-BN/Ta devices. The tunnel barriers (TBs) are compared for electron tunneling at (**a**) V_B_ and (**b**) Ta_B_ devices. The red arrows indicate the width (*d*) and height (*Φ*_TB_) of the TBs, respectively.

Our analysis suggests that the vdW gaps between the Ta electrodes and h-BN monolayer create an energy barrier for vertical electronic emission, resulting in the device initially being in the HRS. In Figure 8a, we can observe two potential barriers at the Ta/V_B_/Ta interface, which we refer to as tunnel barriers (TBs). These TBs are characterized by their width (*d*) and height (*Φ*_TB_), which are determined by the physical separation between the layers and the effective potential (*V*_eff_) peak along the z direction. By analyzing these TBs, we can gain insight into the electron injection efficiency and better understand the essential interface characteristics of Ta/h-BN/Ta that influence its electrical behavior. In Figure 8a, *Φ*_BN_ represents the *V*_eff_ of the monolayer h-BN, while the *Φ*_TB_ is defined as the minimum barrier height that an electron from the metal Ta must overcome if it has the same potential energy as *Φ*_BN_, and *Φ*_Ta_ denotes the minimum *V*_eff_ that an electron can have in the metal Ta. Therefore, *Φ*_TB;L and_ *Φ*_TB;R_ can be calculated as the difference in *V*_eff_ between the left and right vdW gap (*Φ*_gap_) and BN (*Φ*_BN_). The widths *d*_L_ and *d*_R_ are defined as the physical interlayer spacing on both sides. Therefore, the interlayer distances at the Ta/V_B_/Ta device results in large tunneling TBs (*Φ*_TB;L_ = 37.83 eV, *d*_L_ = 3.82 Å and *Φ*_TB;R_ = 37.82 eV, *d*_R_ = 3.82 Å), effectively inhibiting the direct tunneling effect at the vdW interfaces. However, when a full-chain conductive filament is formed on both sides of the interface, the interlayer distances and TBs also decrease significantly, as shown in Figure 8b. Narrow and low TBs (*Φ*_TB;L_ = 29.88 eV, *d*_L_ = 1.81 Å and *Φ*_TB;R_ = 29.89 eV, *d*_R_ = 1.81 Å) can significantly improve the electron injection efficiency, which may be the source of the LRS in the atomristor.

**Figure 9:**
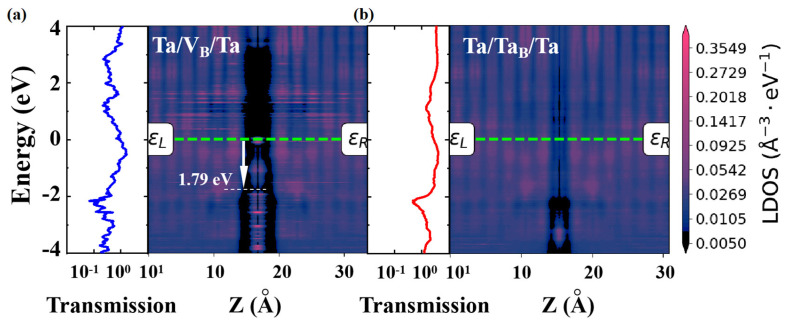
A comparison of the Schottky barriers, band gap states, and transmission coefficient in (**a**) V_B_ and (**b**) Ta_B_ devices. The green dotted lines represent the Fermi level.

Additionally, the electron injection efficiency in devices is closely related to the SB heights and band gap states between the electrode and dielectric layer. These factors can be determined from the projected device density of states (PDDOS) shown in Figure 9. The PDDOS can be projected onto the intermediate h-BN dielectric layer, providing a clear depiction of the distribution of states and band edges in real space. Regions with low (high) DOS are represented by dark (bright) colors, indicating forbidden (permitted) bands within the device. The vertical p-type (n-type) SBs are derived from the energy difference between the Fermi level and the VBM (CBM) of the monolayer h-BN at the left and right interfaces, as illustrated in Figure 9a,b. In the V_B_ device, a p-type SB contact is formed at the interface with Ta electrodes, while an Ohmic contact is formed in the Ta_B_ device with a fully conductive channel. Additionally, the weak interfacial interactions between the Ta electrode and monolayer h-BN with V_B_ defect result in a limited formation of band gap states at the interfaces of the electrode and dielectric layer. Therefore, the vdW gap with a clean interface creates a vertical insulation characteristic in the Ta/V_B_/Ta device, ensuring the HRS. Interestingly, the SB disappears when the Ta atoms are adsorbed above the V_B_ defect or when Ta atoms passing through the monolayer h-BN via the V_B_ defect, as shown in Figure 9b. We observed that the band gap states created by Ta_B_ occur throughout the band gap region of h-BN, allowing for charge flow from the metal into the gap states, resulting in an enhancement of the transmission coefficient [42,43]. Our analysis suggests that both interfacial and atomic defects contribute to these gap states. The covalent bonds of the Ta atom on the V_B_ defect, and the chemical bonding of Ta with the metal electrode, strongly disturb the band structure of the monolayer h-BN, resulting in the disappearance of the band gap in the middle region and barrier-free carrier injection in the vertical direction. Therefore, the band gap states in monolayer h-BN significantly affect and even dominate the device performance.

**Figure 10:**
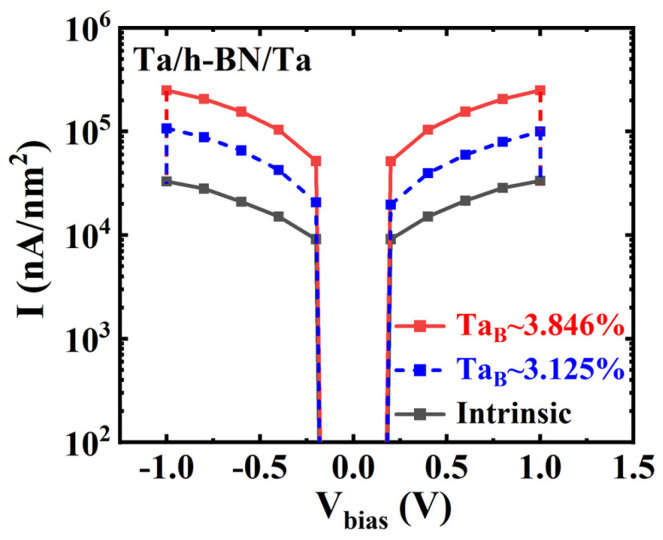
Calculated *I*-*V* curves of Ta/h-BN/Ta atomristors without and with a V_B_ defect of 3.846% and 3.125%, respectively.

Additionally, the size of the device and the density of defects have a significant impact on the resistive switching characteristics of the atomristor. Figure 10 illustrates the *I*-*V* curves of the monolayer h-BN atomristors without and with V_B_ defects, at densities of 3.846% and 3.125%, respectively. It is evident that the current varies with the defect density at the same voltage. This suggests that by controlling the defect density of V_B_, multiple resistance states can be achieved in monolayer h-BN atomristors. Therefore, the presence of a low defect density of Ta_B_ may explain why the device is in the HRS or intermediate−resistance state, while a high density of Ta_B_ can result in an efficient LRS. In addition, we hypothesize that the intrinsic h-BN structure may also contribute to the HRS. At high driving voltages, the V_B_ defects in h-BN can be created via field-induced removal, similar to the removal of S atoms in MoS_2_ [21,44]. This prediction can also explain the varying HRS values observed in experimental devices [23]. Therefore, monolayer h-BN atomristor has the potential to achieve multiple resistance states through a single defect, even at the thickness miniaturization limit.

## 4. Conclusions

To summarize, this study examines six key criteria—adsorption energy, transport current, diffusion barrier, TBs, SBs, and band gap states—to explain the conductive mechanism in the Ta/h-BN/Ta atomristor. The resistance switching behavior can be attributed to the dissociation–adsorption–permeation process of Ta atoms from the electrode onto V_B_ defects. The presence of larger TBs and SBs in the vdW gap between Ta and monolayer h-BN results in an insulating characteristic in the vertical direction. The combination of metal electrode contact and the adsorption of Ta_B_ defects can alter the interface barrier between the electrode and dielectric layer, as well as create band gap states within the band gap of monolayer h-BN. These band gap states can shorten the effective tunneling path for electron transport from the left electrode to the right electrode, resulting in an enhancement of the tunneling current. Our theoretical calculations provide a detailed understanding of the interface and defect properties between the Ta electrode and monolayer h-BN, which ultimately determine their electron transport characteristics.

## Data Availability

The data that support the findings of this study are available from the corresponding author upon reasonable request.
